# Listen to Me! Target Perceptions of Digital Hate: A Scoping Review of Recent Research

**DOI:** 10.1177/15248380241303725

**Published:** 2025-01-04

**Authors:** Maryam Khaleghipour, Kevin Koban, Jörg Matthes

**Affiliations:** 1 University of Vienna, Austria

**Keywords:** scoping review, digital hate, hate targets, victimization, intersectionality, coping

## Abstract

Digital hate is typically targeted toward individuals or groups based on distinct attributes. Despite numerous studies on targets of digital hate, there is a lack of a systematic meta-perspective on targets’ perceptions of digital hate. Therefore, this scoping review aims to assess available definitions and characteristics of targets, consequences of digital hate together with targets’ reactions and coping strategies, dominant methodologies, and identified future outlooks of digital hate victimization research. To achieve this goal, we systematically searched two established databases (i.e., Web of Science and Scopus) for research published from 2020 onwards using a comprehensive search string of digital hate terms. Out of the 12,978 publications screened for eligibility, 230 studies met the inclusion criteria for this review. All selected studies were in English and featured targets of digital hate as the sample. The findings indicate a lack of conceptual clarity, a strong dominance of Global North perspectives, a scarcity of research on children, older adults, men, and people from minority groups, and a need for experimental and longitudinal quantitative research methods, as well as qualitative and mixed-method research. Most importantly, we found that victimization consequences and coping strategies adopted by targets have been researched without sufficiently accounting for inconsistently privileged and intersectional identities and without examining contextual effectiveness. This review, therefore, emphasizes the necessity of taking an intersectional approach to gain a thorough understanding of targets’ digital hate victimization experiences and both short- and long-term coping effectiveness.

It is widely accepted among communication scholars that social media can be a force for good (Castaño-Pulgarín et al., 2021). However, social media affordances such as anonymity and mobility, which can foster public deliberation under some conditions, also serve as catalysts for people to spread digital hate (Brown, 2018; Rothut et al., 2024). Through countless manifestations, digital hate is typically targeted toward people or groups based on distinct attributes such as age, gender, sexual orientation, race, ethnicity, religion, physical appearance, disability, nationality, and socioeconomic status (Celuch et al., 2023), and/or professional status (e.g., politicians, journalists, and scientists; Obermaier, 2023). Experiencing digital hate is associated with serious psychosocial consequences for targets, especially those from minority groups (Keighley, 2022). Likewise, targets from supposedly advantaged groups such as journalists, politicians, or scientists also experienced fear and insecurity that sometimes led to self-censorship or intentions to leave their profession (Lee & Park, 2023). Beyond individual impacts, digital hate also has far-reaching societal consequences as it threatens social cohesion and undermines democratic foundations by radicalizing online environments (Barth et al., 2023) and silencing targets (Celuch et al., 2023).

Extant research has studied digital hate targets extensively; however, despite all this invaluable work, several crucial challenges are still lying ahead. Conceptually, available definitions of targets are vague and inconsistent (Fissel et al., 2022). Overall, it is not clear which people are primarily studied as targets and who defines them (a) as a particular group and (b) as targets in the first place. Here, a broad scoping review might allow for a much-needed comprehensive overview.

Despite these concerns, it is accepted that digital hate victimization can be harmful to all targeted people (Kansok-Dusche et al., 2023) but not necessarily in the same way as targets may individually make use of coping strategies (Wolfers & Utz, 2022). This heterogeneity needs to be made visible to understand better how targets perceive the consequences of victimization and how they respond to them. Importantly, we argue that such research cannot only study targets but needs to integrate their unique perspectives directly. Systematizing available insights into psychosocial digital hate impacts and coping strategies among targets with inconsistently privileged or intersecting identities (e.g., a socially empowered person with a minority identity like, for instance, a Muslim politician in a Christian country or a person who is a part of various minority groups like, for example, a Black disabled woman who identifies as LGBTQIA+) with their own experience with digital hate is needed to advance a holistic research agenda.

A scoping review may constitute an important step in this direction, as it can illuminate general conceptual, empirical, and methodological trends as well as common shortcomings and recommendations within a particular field of study to provide a broad overview concerning the scope of available studies that may consecutively lead to more precise follow-up reviews (Munn et al., 2018). In this particular case, it can provide systematic insights into how recent literature has approached digital hate targets. More specifically, we aim to discuss (a) available definitions and conceptualizations of digital hate targets, and also identify the institutional backgrounds of authors (i.e., professional affiliations with Global North or Global South institutions) who provided these definitions and conceptualization, (b) documented consequences of digital hate together with (c) targets’ responses and coping strategies, (d) dominant and peripheral methodologies present for studying targets, (e) and recommendations for future research identified in the sampled literature.

## Conceptual Heterogeneity Within Digital Hate Research

In recent years, various digital hate phenomena have received much scholarly attention (Barth et al., 2023). Generally, digital hate, when compared to non-digital hate, is shaped by social media affordances such as anonymity, invisibility, and instantaneousness (Brown, 2018) that facilitate production and distribution across different manifestations. Moreover, digital hate is often spread through searchable hashtags, which extends the reach and lifespan of an initial social media post, making it more accessible to a wider audience and potentially affecting more individuals (Kakavand, 2024). Consequently, these aspects of social media can make digital hate even more stressful than non-digital hate, for instance, by making it easier for perpetrators to produce and disseminate hateful content and more stressful for targets by extending exposure duration and collapsing social contexts (Sourander et al., 2010).

Although the literature on digital hate lacks consensus regarding its definition and conceptualization, following Matthes et al. (2023), digital hate is understood here as an umbrella term including any form of digitally transmitted malevolent and hostile expressive behavior targeted toward an individual or a group. While some scholars perceive digital hate as “a heterogeneous collection of phenomena held together by family resemblances” (Brown, 2018, p. 610), reviewing recent research demonstrates a conceptual overlap between various forms of digital hate (despite acknowledging the lack of standardization and poor definitional practice, e.g., see Kansok-Dusche et al., 2023 or Matthes et al., 2023). In general, digital hate is theorized as a form of communication in which individuals or groups are either targeted based on a collective characteristic (e.g., gender, sexual orientation, race, and religion) or otherwise personally attacked, for instance, to threaten or embarrass them (e.g., cyberstalking or online dating abuse; Fissel, 2022). As such, digital hate comprises various established concepts, such as online/cyber hate speech, harassment, hostility, aggression, racism, dating or partner abuse, bullying, stalking, trolling, and other forms of antisocial behaviors (Matthes et al., 2023). It is still unclear what specific phenomena they represent and how they can be distinguished from each other (Barth et al., 2023). Therefore, differentiating targets across these manifestations is inherently problematic.

Considering such a heterogeneous body of literature, a scoping review seems the most appropriate way to clarify key concepts, map available evidence, and identify current research avenues (compared to systematic reviews and meta-analyses; Munn et al., 2018). However, for a scoping review to be most valuable, we argue that it is necessary to avoid further conceptual noise and instead adopt a broad approach. The present scoping review may thus create a comprehensive starting point for studying targets’ characteristics, consequences of digital hate, and idiosyncratic coping preferences that can be used for developing strategies and policies to help and empower affected individuals.

### Conceptualization of Targets

Partially due to conceptual heterogeneity, there is no universally accepted definition of digital hate targets.^1^ Many targets are defined based on their age, gender, sexual orientation, race, ethnicity, religion, physical appearance, disability, and socioeconomic status (Castaño-Pulgarín et al., 2021; Celuch et al., 2023). In general, children and adolescents (Zhu et al., 2021), women (Chen et al., 2020), the LGBTQIA+ community (Schoenebeck et al., 2023), people of color (POC; Keum, 2023), Muslims (Evolvi, 2019), and disabled people (Moral et al., 2022) are considered typical targets from socially non-empowered groups. In recent years, some polarized issues such as the COVID-19 pandemic have given rise to digital hate against societally empowered groups such as politicians, journalists, and academics whose work may include maintaining an online presence and the dissemination of information with broad and diverse online audiences (Farrell et al., 2020). Naturally, empowerment statuses can also co-occur in inconsistently privileged targets who have not yet attracted much scholarly attention.

In addition to studying targets based on societal empowerment status, prior research has also conceptualized targets via dispositional traits (Escortell et al., 2020), showing that basically everyone could be a target of digital hate. A growing body of studies has investigated the role of the Big Five personality traits (see McCrae & Costa, 1997) on victimization and found that especially neuroticism and extraversion may be predictive of being targeted by digital hate (Escortell et al., 2020). Others showed that low levels of self-esteem (Extremera et al., 2018) and self-efficacy (Heiman & Olenik-Shemesh, 2022) are positively associated with being victimized. However, the vast majority of research exploring the relationship between personality traits and digital victimization concerns cyberbullying particularly among adolescents (Peluchette et al., 2015). This focus limits understanding of how dispositional traits might contribute to diverse digital hate concepts across different populations, underscoring the need for broader investigation.

For both demographic- and disposition-driven victimization, intersectional identities have remained underexplored. Intersectionality theory is an analytical framework proposing that individuals’ social and political identities (and groups that are affiliated with or assigned) bring about unique combinations of discrimination and privilege (Crenshaw, 1991). Research on offline harassment indicates that multiple marginalized identities lead to an increased risk of mistreatment, intensifying the impacts on targets (Buchanan et al., 2018). However, in the online environment, research on digital hate tends to oversimplify target groups, often underestimating the diversity of their experiences (Healy, 2019). Therefore, the exploration of inconsistently privileged or intersectional identities is an essential task for conceptualizing digital hate targets. The scoping review allows us to systematically review the conceptualization of targets in recent research, enabling us to identify gaps in this literature. We ask:

**RQ1a:** How are digital hate targets defined and conceptualized in recent research?

It has been highlighted that the vast majority of digital hate authors work in Global North institutions and have focused on target populations from the Global North, resulting in a lack of evidence from other contexts (Livingstone et al., 2016). It has been pointed out that researchers who are privileged to not have been through certain kinds of victimization themselves may accidentally introduce bias because it has never been their lived experience (e.g., Dupree & Boykin, 2021). It is, therefore, essential to also consider the institutional backgrounds of those who conceptualize digital targets, which is why we ask:

**RQ1b:** Who defines and conceptualizes targets of digital hate?

### Consequences of Digital Hate Victimization

Experiencing digital hate as a target often comes with psychosocial or psychopathological harms, such as perceived stress, anxiety, depression, and suicidality (see Tran et al., 2023; Tynes et al., 2020), especially for minorities due to so-called minority stress (see Meyer, 2003). Mismatch and disharmony between an individual from minority groups and the dominant culture can be burdensome, leading to significant stress following experiences of discrimination (McConnell et al., 2018). Aside from individual experiences, vicarious victimization (i.e., group-level attacks where an individual is not directly, but indirectly targeted via affiliation; see Tynes et al., 2020) has also been documented with links to mental health problems, for instance, due to threatening the social identity of an individual indirectly (Portillo et al., 2022; Saleem & Ramasubramanian, 2019). Moreover, the consequences of online victimization cannot be isolated from the offline world where targets may become increasingly concerned for their safety (Logan, 2023). Importantly, these consequences vary by individual, as different targets might perceive the severity of digital hate differently, resulting in varying consequences (Schoenebeck et al., 2023). Thus, we ask:

**RQ2:** What are the consequences of victimization on digital hate targets?

### Coping with Digital Hate Victimization

The transactional model of stress and coping (Lazarus & Folkman, 1984) is considered the main theoretical framework for research on coping strategies. According to this theory, people constantly assess environmental stressors regarding their significance (i.e., primary appraisal) and, if considered significant, determine what they can do to deal with it (i.e., secondary appraisal). Once individuals feel that stressor-driven demands exceed their available resources, they adopt coping strategies to regulate their emotions and restore their fundamental needs. As per Riva (2016), these coping strategies can be clustered along a cognitive-behavioral and an approach-avoidance dimension, fundamentally differing in whether we classify them as silent or non-silent. Specifically, behavioral approach (i.e., activities directly addressing the stressor) most often would be regarded as a non-silent strategy, while behavioral avoidance (i.e., activities unrelated to the stressor), cognitive approach (i.e., concentrating one’s thoughts on the stressor), and cognitive avoidance (i.e., active effort to avoid thinking about the stressor) are silent coping strategies. Thus far, it is unclear how prevalent different coping strategies are across targets. What may be even more crucial is that there is a lack of evidence about coping strategies’ long-term and short-term effectiveness in restoring psychological outcomes and discontinuing online hate (see Khaleghipour et al., 2024; Maran & Begotti, 2022). The scoping review may allow us to systematize coping strategies used by different targets together with insights into their effectiveness. We ask:

**RQ3:** How do digital hate targets react to and cope with victimization?

### Methodological Scope

As interest in digital hate as a research subject rises, methodologies that provide both in-depth and fine-grained target insights are becoming increasingly necessary. Similar to other fields, qualitative data as well as large-scale investigations have been highlighted to be lacking (Smith, 2019). Scholars have called for longitudinal and experimental methods to help explore causality (Thomas et al., 2023), and mixed-method and comparative approaches to avoid biases from single methods and contexts (Maxie-Moreman & Tynes, 2022). Importantly, passive observation methods have typically been preferred over data collection where targets are directly questioned about their victimization experiences (Lingiardi et al., 2020). To obtain an overview of the methodological scope of studying digital hate targets, examining methodologies, and research methods in the recent literature about digital hate targets is needed. Therefore, we ask:

**RQ4:** Which methods are dominant for studying targets of digital hate?

### Shared Future Outlooks

Finally, most publications discuss future outlooks that are informed by research gaps. Systematizing these statements to capture common threads is a key task for this scoping review given that identified future recommendations could open up previously unrecognized synergies. In other words, providing a systematic overview of future research directions can inform us about which research gaps are most prevalent in the field across targets and which have been neglected, which is why we ask:

**RQ5:** What are future outlooks in digital hate victimization publications?

## Method

To address these questions, we conducted a scoping review following the preferred reporting items for systematic reviews and meta-analyses extension for scoping reviews (PRISMA-ScR) guidelines (Tricco et al., 2018). Specifically, we searched electronic databases that are considered reliable using a thorough list of search terms and screened all records to identify eligible studies according to inclusion criteria. Online Supplements can be found at https://osf.io/yd2wx/.

### Search Strategy

To find and select relevant articles, we conducted a Web of Science (WoS) literature search, followed by an additional Scopus search. Two groups of search terms were selected: (1) *digital terms* (to ensure relevance to the digital environment), and (2) *hate terms* (to identify research relevant across hate types). Additionally, we entered the words “target” and “victim” to ensure proper identification. We opted for a second search round, using *digital hate terms* such as “deepfake,” “trolling,” “revenge porn,” and “doxing” that are online per se, such that they might not be paired with digital terms (see Supplemental Appendix, Table A for final search string). We limited our search to articles, proceeding papers, and early access in English and to WoS categories relevant to this review’s topic, including social sciences, communication, psychology, and computer sciences (see Supplemental Appendix, Table B).

Entering this search string, we searched for studies published between January 1, 2020, and July 20, 2023 (i.e., the day of the literature search). We selected this starting date as a compromise between feasibility, relevance, and generalizability, highlighting 2020 as a globally relevant breaking point for how digital hate has spread and, more importantly, has been targeting users. Specifically, the period from 2020 onwards has been marked by various significant events, such as the global COVID-19 pandemic, which increased online activity and potentially heightened instances of digital hate, in general, all over the world, including toward scientists specifically (Farrell et al., 2020; O’Grady, 2022). Additionally, various political events and social movements during this time, such as the 2020 United States elections and the global rise of right-wing authoritarianism, the murder of George Floyd, and ongoing issues such as the European immigration crisis and regional conflicts, have had substantial impacts on fragmenting and polarizing societies, which have been connected with an alarming rise of digital hate on social media (Lupu et al., 2023).

The WoS search yielded 5889 studies to be screened for eligibility. All records were screened by the first author. Screening for eligibility was first based on (1) the article titles. However, since titles sometimes turned out too vague to make a reliable judgment, unclear cases were (2) abstract-screened. On December 1, 2023, an additional Scopus search was conducted to ensure comprehensive coverage. Using the WoS search string (but readjusting according to a Scopus format), only papers published until July 10, 2023, were included, resulting in 7089 records for screening.

### Exclusion Criteria

Given our emphasis on digital hate targets, we excluded all records that (1) focused exclusively on digital hate perpetrators and bystanders as well as (2) on conceptualizations or the content of digital hate (e.g., discourse or content analysis of hate on social media). Several studies focused on more than one actor, for instance, when examining target-perpetrator and target-bystander dynamics. We included these records but only focused on target-related results. Studies about (3) digital intervention for offline hate, (4) child abuse, and (5) hate crime for economic interests were also excluded.

**Figure 1.**
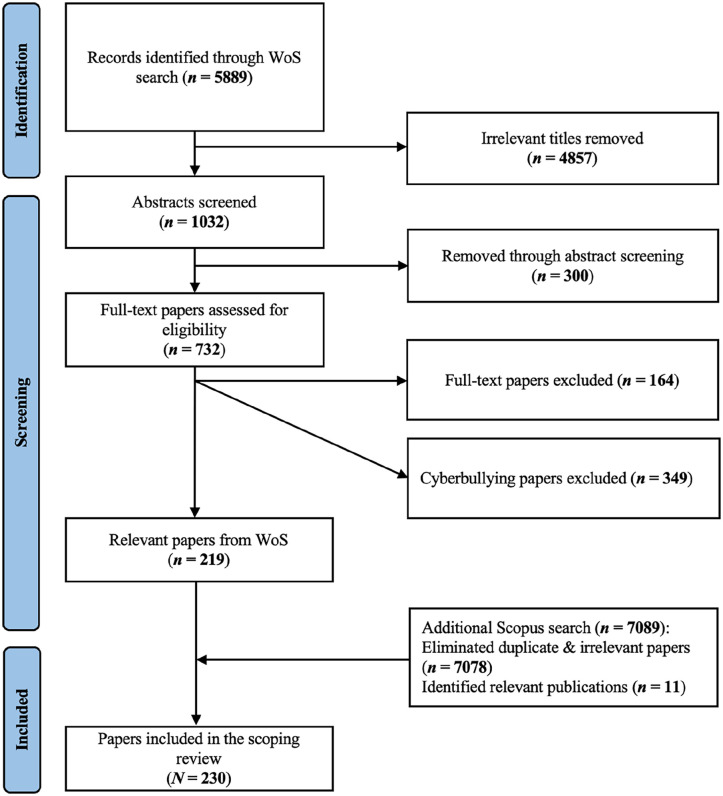
PRISMA Flow Diagram for all Stages of the Literature Search.

Based on this criteria, 5,157 records from WoS search were dropped after title and/or abstract screening, leaving 732 articles for full-text screening. After reading the remaining documents, 164 additional articles that did not account for target perceptions were excluded (e.g., about organizational support for empowered targets, or inter-/prevention for non-empowered targets). While our initial approach was to also include studies on cyberbullying to cover the full breadth of digital hate, we concluded during the screening process that it has already been well-covered via several recent scoping reviews (Yosep et al., 2023a, 2023b). Accordingly, we excluded cyberbullying papers (349 records), which resulted in a sample of *N* = 219. Among the 7089 records retrieved from Scopus, 7076 were duplicates or irrelevant, leaving 11 additional relevant publications, which resulted in a final sample of *N* = 230 (see Figure 1). A full list of citations that were included in our analysis can be found in the Online Appendix.

### Coding Strategy

Each included study was quantitatively and qualitatively coded by the first author for 25 categories. A thorough and detailed overview of the category operationalizations in the codebook can be found in the Supplemental Appendix (Table C). In the quantitative part, we analyzed 18 categories: year, main concept, methodologies, research methods, targets’ age, gender identity, race/ethnicity, religion, refugee status, group status, disability, socioeconomic status, sexual identity, political orientation, personality traits, appearance, as well as authors’ affiliated institution. In the qualitative part, seven categories were analyzed: targets’ geographical allocation, definition of target(s), consequence(s) of digital hate, coping strategies, future outline(s), as well as the country of the authors’ affiliated institution^2^ (see Supplemental Appendix, Table D for both quantitative and qualitative codings). For quantitative categories, we established predetermined category levels, while for qualitative categories, we extracted complete sentences or phrases from the text. To ensure the validity of quantitative coding, two trained coders assessed 23 randomly chosen documents (10% of the sample) based on the established codebook. The coding process yielded sufficient agreement (Krippendorf’s α ≥ .8) across all categories (see Supplemental Appendix, Table E).

## Results

Table 1 displays the frequency of all categories except authors’ institutional affiliations, which are presented in the Supplemental Appendix (Tables F and G). To improve readability, we also provide separate descriptive tables for each category in the Supplemental Appendix.

**Table 1.** Quantitative Coding with Multiple Categories.

Category	Occurrence (*N* = 230)
Year	2020 (*n* = 52; 23%); 2021 (*n* = 36; 15%); 2022 (*n* = 62; 27%); 2023 (*n* = 80; 35%)
Main concept(s)	Cyber/technology-facilitated/digital/online/image-based (sexual) abuse/harassment/violence (*n* = 75; 33%); online/cyber/social media racism/(ethnic-racial) discrimination (*n* = 48; 21%); cyber/ technology-facilitated/digital/online dating/domestic/intimate partner abuse/violence/victimization (*n* = 42; 18%); cyber/online stalking (*n* = 16; 7%); cyber/online/digital hate (speech; *n* = 15; 7%); cyber/online victimization (*n* = 10; 4%); cyber/online exclusion/ ostracism (*n* = 6; 3%); online incivility; cyber/online (micro) aggression (both *n* = 5; 2%); online hostile/toxic behavior (*n* = 3; 1%); cyber/online grooming; revenge porn (both *n* = 2; 1%); trolling (*n* = 1; <1%)
Age	Emerging adults (*n* = 132; 57%); adults (*n* = 76; 33%); adolescents (*n* = 67; 29%); older adults (*n* = 44; 19%); children (*n* = 0); not specified (*n* = 45; 20%); not applicable (*n* = 5; 2%)
Gender identity	Male and female (*n* = 124; 54%); male and female & nonbinary (*n* = 55; 24%); female (*n* = 40; 17%); male (*n* = 5; 2%); not specified (*n* = 6; 3%)
Race/ethnicity	Diverse with White (*n* = 71; 31%); diverse without White (*n* = 22; 10%); Black/African-American (*n* = 13; 6%); Asian (*n* = 7; 3%); Hispanic/Latinx (*n* = 5; 2%); other (*n* = 2; 1%); White (*n* = 1; <1%); Native/ Indigenous (*n* = 0); not specified (*n* = 109; 47%)
Religion	Diverse (*n* = 5; 2%); Islam (*n* = 1; < 1%); Christianity; Judaism; Hinduism; Buddhism (each *n* = 0); not specified (*n* = 224; 97%)
Refugee status	No (*n* = 228; 99%); yes (*n* = 2; 1%)
Targets’ geographical allocation	USA (*n* = 103; 45%); Spain (*n* = 16; 7%); Australia (*n* = 10; 4%); Canada (*n* = 11; 5%); China (*n* = 6; 3%); South Korea; Germany (both *n* = 5; 2%); Finland (*n* = 4; 2%); Chile; Norway; Portugal; Sweden; UK (each *n* = 3; 1%); Belgium; Croatia; India; Iran; Italy; Switzerland; Uganda (each *n* = 2; 1%); Afghanistan; Bangladesh; Brazil; Egypt; Hungary; Israel; Namibia; Nepal; Netherlands; Philippines; Romania; Russia; Sri Lanka; Tanzania; Zimbawe (each *n* = 1; < 1%); multinational (*n* = 23; 10%); no information (n = 3; 1%)
Group status	Non-empowered (*n* = 201; 87%); empowered (*n* = 29; 13%)
Disability	No (*n* = 225; 98%); yes (*n* = 5; 2%)
Socioeconomic status	Diverse (*n* = 38; 17%); low; middle class; high (each *n* = 0); not specified (*n* = 192; 83%)
Sexual identity	Diverse (*n* = 55; 24%); LGBTQIA+ (*n* = 13; 6%); non-LGBTQIA+/heterosexual (*n* = 5; 2%); not specified (*n* = 157; 68%)
Personality traits	No (*n* = 207; 90%); yes (*n* = 23; 10%)
Political orientation	Diverse (*n* = 10; 4%); right-wing/conservative; moderate; left-wing/liberal (each *n* = 0); not specified (*n* = 220; 96%)
Appearance	No (*n* = 228; 99%); yes (*n* = 2; 1%)
Methodology	Quantitative (*n* = 168; 73%); qualitative (*n* = 47; 20%); mixed-method (*n* = 15; 7%)
Research methods	Cross-sectional survey (*n* = 145; 63%); semi-structured interviews (*n* = 31; 14%); longitudinal survey (*n* = 13; 6%); experiment (*n* = 7; 3%); qualitative content analysis (*n* = 7; 3%); focus group interviews (*n* = 3; 1%); eye-tracking; quantitative content analysis; case study; observation; ethnography; mobile experience sampling (each *n* = 0); mixed methods (two or more different methods; *n* = 24; 10%)

### Definition and Conceptualization of Targets (RQ1a and RQ1b)

Regarding targets’ age group, emerging adults were the most frequently studied group (*n* = 132; 57%), followed by adults (*n* = 76; 33%), adolescents (*n* = 67; 29%), and older adults (*n* = 44; 19%). No study considered children as targets of digital hate, likely a consequence of excluding cyberbullying which focuses on children and adolescents (see Zhu et al., 2021). However, insights about children across other digital hate concepts are nonexistent. It must also be noted that all studies including older adults investigated them together with other adults. A primary focus on the elderly is thus lacking.

Regarding targets’ gender identity, most studies included both men and women in their samples (*n* = 124; 54%); nevertheless, gender distributions were often unequal in favor of female participants. Relatedly, some papers only focused on women (*n* = 40; 17%), while only a few papers included only men (*n* = 5; 2%). When nonbinary gender identities were included (*n* = 55; 24%), it was generally done as control variables, and without presenting comparative results. Similarly, a few articles included only LGBTQIA+ people (*n* = 13; 6%), while the majority of research did not indicate targets’ sexual orientation (*n* = 157; 68%). All these patterns suggest a lack of studies on male and, particularly, nonbinary targets or, more generally, the LGBTQIA+ community (Clevenger & Navarro, 2021).

Members of marginalized ethnic groups were also considered targets of digital hate; however, participants’ racial background was not specified in 47% of publications (*n* = 109), followed by diverse sample with predominantly White participants (*n* = 71; 31%), diverse sample without White (*n* = 22; 10%), exclusively Black/African(-American) (*n* = 13; 6%), exclusively Asian (*n* = 7; 3%), exclusively Hispanic/Latinx (*n* = 5; 2%) and other participants (*n* = 2; 1%). One paper included only White people (*n* = 1; < 1%). Furthermore, looking at the targets’ geographical allocation revealed that most sampled studies focused on participants from the Global North, particularly from the United States.

Only very few studies involved refugees and disabled people (*n* = 2; 1% and *n* = 5; 2%, respectively), and only two papers examined people targeted due to their appearance (e.g., weight and hair; 1%). Surprisingly, only a single publication (<1%) investigated religion, and none examined socioeconomic status, or political orientation as predictors of receiving online hatred. These demographic variables were exclusively used as covariates.

Empowered groups such as journalists, politicians, scholars, writers, media professionals, or social media influencers were included in 13% of the sampled papers (*n* = 29). Among those, it is particularly women who encounter sexist comments that assault, marginalize, stereotype, or cast doubt on their mental competence (Walulya & Selnes, 2023). Interestingly, empowered individuals are often targeted either because of their performance or political orientation or based on personal or collective identities, such as having a surname that sounds foreign, identifying as a woman or nonbinary, having a migrant background, belonging to a religious minority, being part of the LGBTQIA+ community, or having distinctive physical appearance features (Harlow et al., 2022; Obermaier, 2023). Again, most evidence focused on empowered groups from the Global North, meaning there is a lack of insights into the Global South’s empowered targets.

Lastly, 10% of sampled research (*n* = 23) addressed targets’ personalities, suggesting that self-control, self-esteem, self-compassion, the Big Five, resilience, anxious attachment, interpersonal flexibility, and the need to belong are traits associated with victimization.

Beyond who is investigated, RQ1b asked who defines and conceptualizes digital hate targets. We categorized authors’ geographical allocation by first extracting where their institutions are located, followed by a quantitative classification in Global North and South (based on a classification by the United Nations Conference on Trade and Development [UNCTAD], 2023; see Supplemental Appendix, Table F & G). Overall, 90% of sampled studies were published by first authors affiliated with Global North institutions (*n* = 206), including 46% (*n* = 106) from the US. Only 10% are led by Global South-affiliated authors (*n* = 24), with Asian institutions accounting for most of them (*n* = 17), followed by African (*n* = 5) and Latin American institutions (*n* = 2). Similar numbers can be found for second authors. Digital victimization literature, thus, confirms a Western bias regarding conceptualizing targets.

### Consequences of Digital Hate Victimization (RQ2)

Concerning the consequences of digital hate victimization, research demonstrates that (almost all) targets experience poor overall well-being and mental health compared to non-targets. Psychosocial and psychopathological outcomes range from stress, anxiety, hopelessness, sadness, anger, uncontrollable distress, depression, shock, and psychological trauma to suicide ideation (e.g., Keum, 2023; Logan, 2023). Furthermore, victimization has been linked to lower levels of self-esteem, self-efficacy, self-control, and feelings of lesser self-worth, as well as feelings of being excluded and rejected by others, which has been connected to loneliness and even suicide attempts (e.g., Thomas et al., 2023). Poor mental health due to digital hate victimization has also been documented to lead to poor physical health, such as weight loss and sleep problems (e.g., Huber, 2023). Importantly, digital victimization can affect offline-world perception as it tends to reduce, for instance, perceived safety in the real-world environment (Logan, 2023). In addition to these short- and long-term intraindividual consequences (e.g., lower same-day and next-day mental health, or a sense of isolation and depression symptoms one year later), reported effects also include harmful interpersonal tendencies such as antisocial conduct and a desire to take revenge (e.g., Wachs, 2022). According to some studies, target-perpetrators experience more harmful outcomes than targets who do not also engage in perpetration (Schokkenbroek et al., 2022).

Studies further indicate that vicarious victimization which involves one’s social groups has a stronger negative impact on life satisfaction and loneliness than witnessing outgroup attacks while being less severe than direct victimization (Stahel & Baier, 2023). In other words, when people from non-empowered groups (e.g., POC, the LGBTQIA+ community, and women) witness online discrimination against ingroup members, they also feel vulnerable (Liby et al., 2023; Williams, 2021). Importantly, historical and social circumstances (e.g., concerning anti-Black violence, heteronormative and misogynistic societies, or the COVID-19 pandemic) can significantly impact how minority people perceive digital hate victimization and increase psychological harms for Black people, the LGBTQIA+ community, women, and Asians, respectively (Kamp et al., 2022; Keighley, 2022). Only a few studies have examined digital hate victimization among individuals with intersectional identities, revealing that the combination of multiple marginalized identities exacerbates negative victimization consequences. For instance, women of color may experience a heightened sense of racial trauma, fear, and anxiety compared to men of color (e.g., Williams, 2021). Similarly, women belonging to the LGBTQIA+ community may report a greater loss of trust compared to their male counterparts (e.g., Keighley, 2022).

Digital hate victimization can further happen to empowered individuals (Björkenfeldt, 2023). Here, research also indicates that victimization comes with psychosocial harms (such as distress, anxiety, lower trust, and a sense of alienation). It is also documented that it can negatively affect their willingness to work and may lead to professional detachment or self-censorship (e.g., Obermaier, 2023). For example, journalists who have experienced digital hate have been found to avoid topics that they think will result in backlash, influencing the variety and quality of publicly available information (Holton et al., 2023).

Finally, regarding evaluations of perceived harm, only a few studies showed substantial differences across targets. For example, women, older adults, POC, and the LGBTQIA+ community tend to perceive greater harm in victimization (Schoenebeck et al., 2023). Notably, how perpetrators and targets are related to each other influences harm evaluations, even though this can vary (Adams et al., 2022; Fissel, 2022; Liby et al., 2023). One reason for these varying results could be that many studies only looked at hate type but not at targets’ identities and other socially relevant factors. Little is known about how inconsistently privileged or intersecting identities may experience harm (Keum, 2023).

### Coping Strategies Against Digital Hate Victimization (RQ3)

Concerning coping strategies, the vast majority of research looked at coping strategies without a structured approach, highlighting strategies separately rather than grouping them into clusters. A strong body of evidence indicates that targets extensively employ what we call silent coping strategies, including behavioral avoidance strategies (e.g., writing their feelings down, listening to music, drinking alcohol, binge or unhealthy eating, taking illegal drugs or prescription drugs, and turning down new career opportunities), cognitive approach strategies (e.g., rumination and internalizing the stress), and cognitive avoidance strategies (e.g., ignoring the situation and normalization of such hate within their profession; see, for example, Adams et al., 2022; McInroy et al., 2023). Some targets have been documented to use media for behavioral avoidance coping by devoting themselves to mobile phone use (e.g., online gaming and cyberloafing; see Zhou et al., 2022).

Researchers often propose that targets should use behavioral approach strategies with or without utilizing media as a coping tool. Substantial evidence suggests that targets primarily seek help from friends and family compared to talking with a therapist or mental health counselor (Liby et al., 2023). Within media environments, targets reportedly utilize cybersecurity solutions such as blocking and reporting to create safe digital environments (Adams et al., 2022). Additionally, they sometimes produce digital content to speak out about victimization, which can foster meaningful connections and reactions from friends and followers, helping them feel visible, and understood, and boosting their self-esteem and resilience. Few targets prefer talking face-to-face or through social media with perpetrators to defend themselves or persuade them to stop harassing (e.g., Gatwiri & Moran, 2023).

Instead of engaging media, intentional disengagement could be viewed as a coping strategy for balancing the benefits and drawbacks of digital media. Few studies proposed that targets may spend less time online and distance themselves from platforms in which future hate might occur. Relatedly, only a few targets leave social media altogether for a long time (e.g., a few months or a year; Maran & Begotti, 2022).

It is rarely emphasized that coping strategies may differ across targets. Women, LGBTQIA+ individuals, and people of racial minorities are more likely to seek out professional help instead of informal help from friends (Mumford et al., 2023). Others have suggested that personality traits could influence targets’ decision to utilize avoidance or approach coping strategies. A few papers demonstrated that the perceived absence of family, organizational, and/or legal protection leads targets to use silent coping strategies.

Lastly, a crucial aspect missing from the literature concerning coping strategies was their effectiveness in both the short- and long-term. Only a single paper presented targets’ viewpoints, suggesting that coping skills serve as a temporary remedy and that higher-level interventions are required to eliminate digital hate (Liby et al., 2023). Additionally, some researchers highlighted that silent and disengagement coping strategies might temporarily move away targets from distress, but possibly worsening impacts over time. Nevertheless, research evaluating targets’ perceptions concerning the effectiveness of various strategies in reducing psychological harm and discontinuing digital hate is noticeably lacking.

### Methodological Scope (RQ4)

With regard to the research methodology, researchers most often use quantitative methods (*n* = 168; 73%) compared to qualitative (*n* = 47; 20%) or mixed methods (*n* = 15; 7%). Examining methods showed that cross-sectional surveys are employed most frequently (*n* = 145; 63%), followed by semi-structured interviews (*n* = 31; 14%), mixed methods (*n* = 24; 10%) and longitudinal surveys (*n* = 13; 6%). Qualitative content analysis (*n* = 7; 3%), experiments (*n* = 7; 3%), and focus group interviews (*n* = 3; 1%) were conducted rarely.

### Future Outlooks (RQ5)

Several commonly formulated recommendations for future research could be identified. First, there is a consistent call for a greater variety in age groups. Specifically, studies repeatedly concluded that more research is needed with samples of older age groups, particularly those above 65 years. Second, the dominant presence of female participants is said to constrain generalizability toward males as well as gender minorities. Third, studies consistently bemoaned a lack of longitudinal and experimental studies and qualitative and mixed-method research. Fourth, coping behaviors are criticized for being applied in a rather broad manner, whereas more precise categories are considered beneficial. Fifth, a lack of nuance is highlighted, that is, it is stated that only little is known about how targets with varying identities cope with digital hate on different platforms. Finally, research calls for cross-cultural and comparative studies with larger and more diverse samples to determine how overlapping identities contribute to or aggravate marginalization experiences.

## Discussion

The goal of this scoping review was to systematically synthesize the most recent empirical research on target perceptions of digital hate. Reviewing 230 studies revealed that generally speaking, young adults, women, POC, and LGBTQIA+ individuals are most often considered potential targets of digital hate. The vast majority of research, however, tends to sample young adults, White people, women, heterosexuals, and non-disabled people. At the same time, they suggest that a more diverse sample is required to study digital hate victimization. Moreover, we found that outside of cyberbullying research, children under the age of 12 are nonexistent in the recent literature. This absence contrasts sharply with minority participants’ understanding that their vulnerability to digital hate is shaped throughout childhood by seeing or hearing about (online) hate toward ingroup people (Williams, 2021). In other words, children may at least experience vicarious victimization, which can have an impact on their mental health and overall resilience. Previous research also indicates an increase in digital hate directed at refugees (Arcila-Calderón et al., 2022), Muslims (Evolvi, 2019), people with disabilities (Moral et al., 2022), or non-normative appearance (e.g., overweight; Lessard & Puhl, 2022), and political orientation (see Kakavand, 2024). Although media frequently covers topics like racism, Islamophobia, or ablism, we often lack a deep enough understanding of the perspectives of those affected. Future research should not continue to overlook these marginalized communities, as understanding their first-hand experiences is crucial for developing effective support and intervention strategies.

A large majority of leading authors in our sampled literature were affiliated with Global North institutions, especially in the US. This aligns with a notable predominance of targets from the Global North, where the sampled minority groups are also typically from. As a result, targets’ experiences within the English literature are conceptualized based on cultural, religious, and social contexts that are very similar to each other, potentially differing from Global South experiences. This Global North dominance and a higher share of US institutions have been noted in the field of communication and studies on online hate in general (Freelon et al., 2023; Waqas et al., 2019). Yet, the ways digital hate is expressed, and perceived, and its impacts can differ significantly in the Global South due to cultural, and sociopolitical factors, and varied anti-hate regulations (Gagliardone et al., 2015).

Relatedly, people from minority groups (e.g., the Black community in the United States) or even empowered targets (e.g., journalists) are typically considered a homogenous group, despite their various cultural, racial, religious, and socioeconomic origins (see Portillo et al., 2022). An intersectional lens, which considers multiple identities, is lacking in studies of both non-empowered and empowered targets. While the intersectional approach was originally developed to study non-empowered groups with multiple marginalized identities (see Crenshaw, 1991), we found that combinations of non-empowered and empowered identities (i.e., inconsistently privileged identities) have remained underexplored. Multiple intersecting identities can intensify the impacts of digital hate by shaping unique experiences of harm (Keum, 2023) and increasing the tendency to use silent or avoidant coping strategies, such as withdrawing from the online environment (see Keighley, 2022). Future studies should avoid prioritizing a single aspect of a person’s identity when addressing digital hate victimization but rather consider multiple identities that contribute to targets’ experiences.

Viewing digital hate victimization through a broader lens also acknowledges the roles that historical and socio-environmental conditions play in victimization experiences (Crenshaw, 1991). Even if an isolated incident seems harmless to outsiders, marginalized targets might feel that their experiences, or those of their close ones, in racially divisive or LGBTQIA+ hostile environments, have heightened their sensitivity to such incidents and affected the harm caused (Harris & Woodlock, 2022). On a similar note, empowered targets also mentioned that the perceived absence of legal protection partly influences the way that they perceive and cope with victimization (e.g., using silent coping strategies; Björkenfeldt, 2023). Future research should consider these historical, social, and legal layers for gaining more comprehensive insights into digital hate victimization experiences. This is because such incidents are not solely related to the individual level given that the environment surrounding targets also plays an essential role in shaping their perception of victimization.

To deal with digital hate victimization, targets from both non-empowered and empowered groups utilize a variety of coping strategies (including media use; Wolfers & Utz, 2022), with most being inherently silent. Thus far, it is unclear why many targets choose to remain silent about the victimization experience. Moreover, it has likewise not been investigated how people’s implementation of different coping strategies may differ based on inconsistently privileged or intersectional identities and with respect to (perceived) historical, social, and legal circumstances. While just a few research stated that avoidance or silent coping strategies might help targets manage immediate emotional fallout and rebuild resources in the short term, they are pessimistic about their long-term effectiveness (Maran & Begotti, 2022). However, targets’ perceptions of the short- and long-term effectiveness of different coping strategies is a research gap that should be addressed.

An overemphasis on quantitative methods, compared to qualitative methods as well as mixed analytical approaches, can produce misleading evidence, underestimating unique characteristics of individuals and their specific contexts while favoring de-contextualization and generalization (Yilmaz, 2013). This analytical imbalance could be exacerbated when it aligns with prevailing trends in quantitative research methodologies where a preference for cross-sectional surveys over experimental and longitudinal designs has been identified. This preference mirrors the gaps identified in our current scoping review concerning the lack of causality and directionality in understanding why some individuals receive hate, as well as the long-term consequences and effectiveness of coping strategies.

Interestingly, these methodological shortcomings were also the most frequent recommendations made by the studies reviewed. Additionally, studies emphasized the need to include more diverse samples and underrepresented groups such as older individuals, men, and people from marginalized groups. Research also pointed out the importance of considering different target identities to have a more comprehensive understanding of their victimization experiences and coping strategies. Finally, researchers are aware of some limitations, but our review reveals a notable lack of recognition of other equally significant research gaps in this field. Addressing these overlooked areas could provide valuable insights into the broader picture of victimization experiences and coping mechanisms.

### Limitations and Implications

As with any study, our scoping review is subject to limitations. First, although our search covered two major databases and included a comprehensive review of the literature, incorporating additional databases might have yielded more relevant papers. Aside from this, we also acknowledge that we may have missed a few articles due to indexing delays and updates. Second, we only included studies published in English, which may be the lingua franca of the current worldwide academic discourse but biases findings considerably. While the review covers diverse topics and researchers worldwide, it disregards a rich body of non-English language work. Third, we only considered research on target perceptions of digital hate victimization. While this approach provided an overview from the perspective of those targeted, it excluded valuable studies, such as discourse analyses of digital hate in social media posts and comments directed at specific individuals or groups. Finally, our limited scope prevented us from exploring the relationships between targets and other actors (i.e., perpetrators and bystanders), which could offer insights into how these interactions influence targets’ experiences of victimization, perceived harm, and coping strategy selection.

In terms of primary implications, our findings indicate that most individuals victimized by digital hate try to cope with it on their own, while some prefer to cope silently. To support these silent targets, it is crucial to establish safe and anonymous online support systems that offer psychological comfort and potentially encourage them to speak up about their experiences. Additionally, tailored programs should be developed to address the unique needs of diverse individuals, especially those with intersecting identities, and to mitigate the harms of digital hate victimization.

Beyond individual efforts, the role of digital platforms, institutions, and policymakers is crucial. Social media platforms increasingly use algorithm-based content moderation to handle hateful content, but perceptions of what constitutes online hate can vary widely based on individuals’ social identities and cultural backgrounds. To allow for inclusive and heterogeneous public spaces, social media companies may benefit greatly from incorporating target perspectives more effectively when refining content moderation strategies, ideally making them less vulnerable to perpetrators circumventing moderators via subtle codes and dog-whistling (i.e., indirect or coded language, symbols, or references, conveying hateful messages, often targeting marginalized groups without using overtly banned terms). Furthermore, our scoping review indicated that some empowered and non-empowered individuals believe there is a lack of resources and legal protection for cyber victimization (Clevenger & Navarro, 2021). This perceived absence of legal protection can lead to underreporting of victimization and normalization of harassment (Björkenfeldt, 2023), meaning that new policies that make it easier for digital hate targets to report incidents and receive support may need to be introduced and implemented.

## Conclusion

The primary goal of this scoping review was to systematically evaluate the most recent empirical studies on target perceptions of digital hate. We documented a strong dominance of Global North perspectives, resulting in a relative absence of evidence from other geographical and cultural contexts. Sampled literature also showed both a critical scarcity of research on children, older adults, men, and people from various sexual, racial, and religious minorities and a need for experimental and longitudinal quantitative research methods, as well as qualitative and mixed-method research. Most notably perhaps, we found that victimization consequences and coping strategies adopted by targets have primarily been studied without sufficiently taking into account inconsistently privileged and intersectional identities and their contextual effectiveness of coping strategies. Together, we call the field to consider intersecting historical, social, and legal layers combined with intersectional identities to gain a comprehensive understanding of targets, their experiences of digital hate victimization, and short- and long-term coping effectiveness.

**Table:** Critical Findings.

• Critical Findings of Reviewed Literature
• Targets of digital hate are most conceptualized within the context of Global North
• Research related to digital hate victimization is predominantly quantitative
• Children, older adults, men, disabled individuals, and people from various sexual, racial, and religious minorities are underrepresented
• Potential variability in inconsistently privileged and intersectional target identities remains underexplored with regard to perceived harm, psychosocial consequences, and coping strategy effectiveness
• There is a lack of consideration of historical, social, and legal layers for understanding targets’ victimization experiences

**Table:** Policy, Practice, and Future Research Implications.

Focus	Implications
Policy	• New policies need to be legislated to protect targets of digital hate, making it easier for them to report incidents and receive support
Practice	• Safe and anonymous online support should be further established
	• Tailored programs should be developed for digital hate targets, particularly for those with intersecting identities
	• Social media companies need to more effectively incorporate target perspectives when improving content moderation strategies
Future research	• Consider qualitative approaches to gain deeper insights into the targets’ perceptions
	• Involve digital hate targets with diverse empowerment statuses and intersectional identities more actively in the research process
	• Investigate the nuanced consequences of digital hate experienced by various targets
	• Explore short- and long-term effectiveness of coping strategies, ideally with special attention to so far widely overlooked and massively undertheorized silent strategies

## Notes

1. Our preferred term in this scoping review is targets. We avoid using the term victims due to it potentially being in itself victimization.

2. Since our primary goal was to capture who conceptualized targets, we only coded the first and the second authors as leading authors for publications with more than two authors.
